# Potential Mechanisms of Probiotics Action in the Prevention and Treatment of Colorectal Cancer

**DOI:** 10.3390/nu11102453

**Published:** 2019-10-14

**Authors:** Marta Molska, Julita Reguła

**Affiliations:** Institute of Human Nutrition and Dietetics, Poznan University of Life Sciences, Wojska Polskiego St. 31, 60-624 Poznan, Poland; marta.molska@up.poznan.pl

**Keywords:** lactic acid bacteria, mechanism of probiotic action, anticancerogenic action of probiotics, therapeutic dose, therapy and protective effects

## Abstract

Colorectal cancer is one of the most common and most diagnosed cancers in the world. There are many predisposing factors, for example, genetic predisposition, smoking, or a diet rich in red, processed meat and poor in vegetables and fruits. Probiotics may be helpful in the prevention of cancer and may provide support during treatment. The main aim of this study is to characterize the potential mechanisms of action of probiotics, in particular the prevention and treatment of colorectal cancer. Probiotics’ potential mechanisms of action are, for example, modification of intestinal microbiota, improvement of colonic physicochemical conditions, production of anticancerogenic and antioxidant metabolites against carcinogenesis, a decrease in intestinal inflammation, and the production of harmful enzymes. The prevention of colorectal cancer is associated with favorable quantitative and qualitative changes in the intestinal microbiota, as well as changes in metabolic activity and in the physicochemical conditions of the intestine. In addition, it is worth noting that the effect depends on the bacterial strain, as well as on the dose administered.

## 1. Introduction

**Figure 1:**
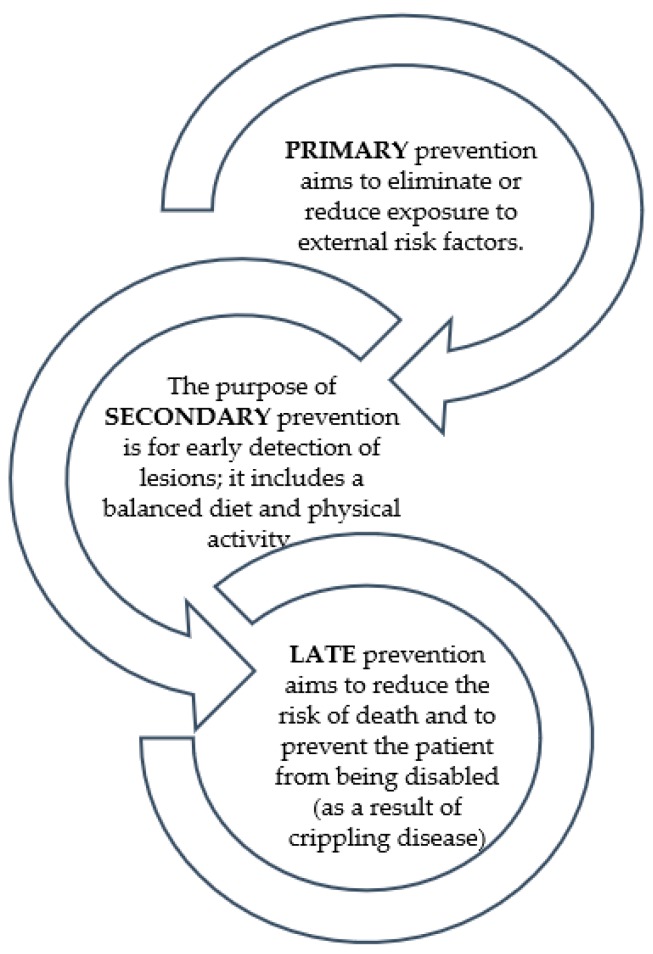
Types of cancer prevention [3,4].

Cancer is a disease that is characterized by the uncontrolled division and survival of abnormal cells. When these changes occur in the rectum or colon, they are referred to as cancerous changes in the large intestine. The incidence of this disease is currently very high and the number of cases continues to rise. Global statistics show that in women colon cancer is second to breast cancer and in men, it is third after lung and prostate cancer [1,2,3]. There are three types of prevention: primary, secondary, and late (Figure 1) [3,4]. Probiotic bacteria may support each stage of prevention. 

Probiotics are defined as “live microorganisms which, when administered in adequate amounts, confer a health benefit on the host” [5]. They include bacteria belonging to the natural intestinal microbiota [6]. Most probiotics belong to the natural intestinal microbiota. They are mainly lactic acid bacteria (LAB), such as gram-positive: *Lactobacillus, Leuconostoc, Lactococcus, Carnobacterium, Enterococcus, Pedicoccus, Streptococcus, Tetragenococcus, Vagococcus, Oenococcus, Weisella,* and *Bifidobacterium* [6,7], as well as *Saccharomyces, Bacillus,* and *Escherichia coli* [7]. It should be emphasized that not all bacteria from a particular species have the same properties and will show the same effect in the organism. Everything depends on the strain and not every strain is a probiotic strain [8]. 

An example of probiotic species/strains with documented characteristics [7,9,10,11,12,13,14]:

- *Lactobacillus casei* Shirota has an inhibitory effect on colorectal cancer and bladder cancer. It exhibits positive effects on maintaining the balance of intestinal microbiome, and also protects against intestinal disorders. It has immunomodulatory effects and can strengthen the immune defense of the host by inducing IL–12 (interleukin 12) production through phagocytes. It supports the treatment of colorectal cancer, decreases the activity of the fecal enzymes, and protects against mutagens from food,
- *Lactobacillus fermentum* NCIMB 5221 is potentially able to modulate hyperinsulinemia, insulin resistance, hypercholesterolemia, and hypertriglyceridemia. It has an antiproliferative effect,
- *Weissella cibaria* JW15 strengthens the function of the immune system by increasing the activity of NK cells (Natural Killer Cells),
- *Saccharomyces cerevisiae* var. *boulardii* has anti-inflammatory and antibacterial effects. It increases the secretion of immunoglobulin A (IgA) and maintains the integrity of the epithelial barrier. It helps in the treatment of travelers’ diarrhea.

The main habitat of bacteria is the intestines. For this reason, it is important for bacteria to reach this part of the digestive tract while still in a living form. Therefore, the bacterial resistance to the action of digestive enzymes or low acidity of gastric juice seems to be necessary [8]. Through their antibacterial properties, probiotic bacteria reduce the growth and adhesion of pathogenic bacteria to the epithelial cells. They compete with other microorganisms for nutrients. This is referred to as direct interaction. There is also an indirect action, which depends on the production of antimicrobial activity compounds, for example, bacteriocin [8,15].

Probiotics are able to influence the qualitative and quantitative composition of the intestinal ecosystem [16]. 

In connection with the above, the purpose of the work is to characterize the potential mechanisms of probiotics, in particular in the prevention and treatment of colorectal cancer, and to present information available in selected literature about probiotics and the potential therapeutic dose. 

## 2. Gastrointestinal Microbiota

The colonization of the human body through a diverse microbiota, as well as the creation of a balanced and diverse ecosystem, is a specific process that requires a great deal of time. The gastrointestinal ecosystem is created from the moment of birth and changes throughout the course of life. There are indications that already in the prenatal period, the microbiome begins to shape [17,18].

It is influenced by various modifiable (for example, diet, antibiotics) and non-modifiable factors (age, sex). The effects of their interaction may initiate colon tumors or inflammatory bowel diseases such as irritable bowel syndrome (IBS). This is a diverse ecosystem which contains more than 10^12^ colony-forming unit per gram (CFU/g) content belonging to about 1000 microorganism species. Colon colonization by bacteria has a large impact on metabolic and enzymatic potentials. Bacteria participate in the metabolism of many endogenous and exogenous compounds. Due to the bacterial activity, many compounds are formed that affect the host’s physiology in a beneficial or harmful way [16,17,19]. The number and the genus of bacteria in the gastrointestinal tract (GIT) are summarized in Table 1.

### Functions of the Intestinal Microbiota

In addition to changes in the profile during human life, the microbiota of the digestive tract also performs important functions in the body: metabolic function, which consists in the production of selected B vitamins, vitamin K, digestion, and fermentation of undigested food residues, energy storage in the form of short chain fatty acids (SCFA), among other things; trophic function is based on the homeostasis of the immune system, as well as control of the intestinal epithelium; and a protective function that is associated with degradation and competition for place in the intestine with pathogenic bacteria [17].

Bacteria belonging to the genus Lactobacillus and Bifidobacterium show an anti-inflammatory effect in the intestine. Reduction of these bacteria may contribute to a low degree of inflammation in which the level of proinflammatory cytokines (e.g., interleukins 6 and 8; tumor necrosis factor α) is elevated in the systemic circulation of patients [24].

## 3. Mechanisms of Probiotic Actions for Reducing the Risk of Colorectal Cancer (CRC)

**Figure 2:**
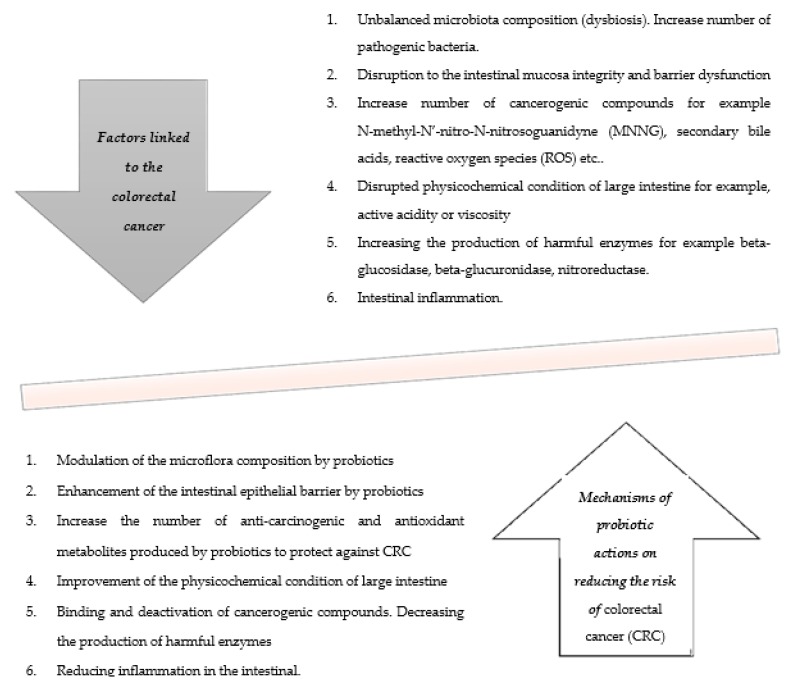
Potential mechanisms of probiotics action and factors related to CRC (colorectal cancer).

There are several mechanisms by means of which the risk of colorectal cancer can be decreased (Figure 2). Based on these, the conclusion is that the prevention of this process is associated with favorable quantitative and qualitative changes in the intestinal microbiota, as well as changes in metabolic activity and in the physicochemical conditions of the intestine [16,25].

### 3.1. Modulation of the Microbiota Composition by Probiotics

First, the composition of the microbial composition can have an influence on the creation of favorable conditions for developing CRC. There should be eubiose (significantly more bacteria of preferred beneficial activity than pathogenic) in the human intestine. When the situation is reversed, there is dysbiosis, which can cause problems with the functions and compositions of intestinal micobiota. As a consequence, chronic inflammation may occur, an increase in the production of cancerogenic compounds, which in turn can exacerbate the risk of CRC [25,26].

Sobhani et al. published results in which they compared samples of feces from healthy people and CRC patients. Their research shows that the number of *Bacteroides* and *Prevotella* genus were significantly higher in the CRC group [27]. In the intestinal ecosystem, several species of the *Lactobacillus* type were present in the intestinal ecosystem in lower amounts than bacteria of the genus *Bacteroides, Eubacterium, Fusobacterium, Prevotella, Proteobacteria*. It was also found that several species of the genus *Salmonella* and *Clostridium* were present in greater numbers in patients with CRC [28]. 

*Bacteroides spp*. and *Clostridium spp*. are classified as bacteria which are involved in the pathogenesis of CRC. In contrast, microorganisms belonging to lactic acid bacteria have been shown to have preventive/protective effects. Probiotics compete with putrefactive and pathogenic bacteria, decreasing their quantity and increasing the number of the LAB bacteria [27,29]. 

*Bacteroides fragilis* produces enterotoxigenic toxin—BFT (fragilysin). This is a compound which increases the risk of CRC. Wnt signaling pathways play an important role in the regulation of processes, including cell differentiation and survival. BFT toxin activates the Wnt signaling pathway dependent on beta–catenin and the nuclear transcription factor kappa B (NF–kB) to increase cellular proliferation. This toxin affects the induction of inflammatory mediators, which leads to the progression of cancer. In addition, it was found that the BFT gene may be one of the risk factors for CRC. It is also particularly associated with its late stage [30,31,32,33]. 

*Fusobacterium nucleatum* is found in larger numbers in people with CRC. This can contribute to progression from adenoma to cancer. Kostic et al. showed the presence of that bacterial species in colon adenoma. Bacteria influenced the increase in the number of tumors and selectively selected tumor-infiltrating myeloid cells. This, in turn, led to tumor progression in an APC^MIN/+^mouse (with a heterozygous APC gene) [34,35,36,37]. Selected bacterial species belonging to *Fusobacterium* influence the immune response on the host. They exhibit virulence traits, promoting the ability to adhere to host cells and penetrate inside. There is a suspicion that *Fusobacterium nucleatum* may be one of the biomarkers for CRC [33,35,36]. 

*Escherichia coli*, with the acquisition of virulence factors, is divided into four phylogenetic groups (A, B1, B2, and D). Groups B2 and D are usually pathogenic (they are involved in parenteral and intestinal diseases). Some strains from the phylogenetic group B2 are associated with chronic inflammatory bowel disease (for example, Crohn’s disease), which poses a risk of CRC [38]. One study demonstrated number of cyclomodulins produced by *Escherichia coli* (from the phylogenetic group B2) on the colon membrane in people with CRC [39]. The pathogenic strain of *Escherichia coli* can synthesize several toxins, for example, cytotoxic necrotizing agent (CNF), cytolethal distending toxins (CDT), and other various virulence factors. Cyclomodulin is a genotoxic toxin. It can be a modulator of cell cycle progression, cell division, and apoptosis [38,39].

*Streptococcus gallolyticus*, *Enterococcus faecalis* can also be connected to CRC [33]. 

Probiotics in an organism affect other microorganisms through their antibacterial properties, the ability to adhere to the epithelium, and antibacterial properties, as well as competition with pathogenic bacteria for space on it. They may bind pathogens or compete with them for nutrients. Probiotics microorganisms were found to produce antimicrobial substances such as bacteriocins, deconjugated bile acids, reuterin, hydrogen peroxide, and lactic acid. Each of these substances can be one of the elements by which probiotic microbiomes inhibit carcinogenic and pathogenic microbes [7,28,40].

### 3.2. Enhancement of the Intestinal Epithelial Barrier by Probiotics

Disruption to the intestinal mucosa integrity and barrier dysfunction results in increased permeability for allergens, leading to an immune stress response and inflammation [41]. The inflammatory reaction is initiated at a particular site and the mucosa adjacent to it. If the pathogenic bacterium enters the intestinal epithelium, it causes damage to the epithelial barrier, increasing the risk of CRC [28]. 

In normal circumstances, the barrier function of the intestine can protect the intestinal tract against toxins, pathogens, and other damage. The full intestinal mucosal barrier includes physical, immunological, chemical, and biological barriers [42]. 

Signaling pathways of epithelial cells are stimulated by whole microorganisms, their structural components, and the metabolites produced by them [28,43]. 

Hsieh et al. showed that some bacterial species of the *Bifidobacterium* genus have the ability to promote epithelial integrity and to prevent disruption of the epithelial barrier induced by TNF–α (tumor necrosis factor α) [44]. In an *in vitro* model of necrotizing enterocolitis (NEC), the researchers showed that probiotic *Lactobacillus* strains strengthen the integrity of tight junctions and the intestinal barrier [45].

Tight junction proteins, claudin–3 and occludin, play an important role in intestinal permeability. One study has shown that the initial treatment of *Lactobacillus rhamnosus* GG and *Lactobacillus reuteri* ZJ617 contributes to a reduction in oxidative stress and inflammation, which in turn leads to improved expression of the tight junction protein, thereby restoring barrier function [46].

Consuming probiotic bacteria can affect the rebuilding of the epithelial barrier by preventing the rearrangement of proteins entering tight junctions and increasing the production of mucus defensins through goblet cells. Additionally, it reduces leakages of harmful substances of dissolved microorganisms and antigens [28,41,47]. 

### 3.3. Increase the Number of Anti-Carcinogenic and Antioxidant Metabolites Produced by Probiotics to Protect Against Colorectal Cancer (CRC)

Probiotic bacteria increase the fermentation of dietary fiber and the level of anti-cancer compounds with therapeutic potential and the activity against CRC, for example, short fatty acids (SCFAs), conjugated linoleic acids (CLAs) and phenols [25,28]. 

SCFAs are end products of the bacterial fermentation of undigested carbohydrates from the diet and endogenous compounds such as mucus. These are organic acids consisting of 1–6 carbon atoms, for example, butyric, acetic, or caproic acid, depending on the location in the large intestine, and the amount of SCFAs produced changes. Moreover, the type of acids produced depends on the substrates supplied from diet [48,49]. Consequently, the production of SCFAs (for example, butyric acid) is associated with diet, chemical composition of digested carbohydrates, intestinal microbiota composition, and the presence of other metabolites. These acids are a source of energy for colonocytes and promote acidosis and apoptosis of cancer cells, thus promoting an acidic environment that inhibits the formation of high levels of secondary bile acids. *Lactobacillus fermentum* NCIMB 5221 and *Bifidobacterium lactis* increase the production of these acids [10,25,28,48]. 

A large amount of probiotics is produced from the lactic fermentation—phenols with antioxidant properties and bioactive fatty acids, such as conjugated fatty acids (CFAs), a group of linoleic acid isomers which have anti-inflammatory and anti-carcinogenic features [25,28]. 

Butyric acid helps regulate the balance between proliferation, division, and apoptosis of colonocytes. Most butyrate (70–90%) is metabolized via colonocytes (for which it is a source of energy). There are indications suggesting the occurrence of that acid in the feces of healthy people in larger amounts compared to CRC patients. In addition, it is assumed that a reduction in the 1µg/L butyrate concentration in stools increases the risk of colon cancer by 84.2%. However, when the concentration of acetic acid is reduced by 1µg/L, the probability of developing adenoma increases by 71.3% [25,48,50]. The concentration of this acid gradually decreases as the stomach contents move towards the descending part of the large intestine, where it is absorbed by colonocytes. Production of butyrate in that part of the intestine is small, because of the low availability of substrates from food [25]. 

Butyrate is produced by a number of bacteria of the genus *Clostridium, Fusobacterium, Eubacterium, Coprococcus* spp., and *Roseburia* spp. Most effectively, this compound is produced by: *Clostridium leptum, Faecalibacterium prausnitzii, Roseburia* spp. and *Coprococcus* spp. It is worth noting that LAB do not produce this compound. However, there are bacterial species that are capable of converting acetate and/or lactate into butyrate (*Eubacterium hallii, Eubacterium limosum,* and *Anaeripes caccae*) [25,48].

Butyric acid is the main energy substrate for colonocytes. Through increased production and proliferation of healthy cells, butyric acid is connected with the enhancement of the intestinal barrier. This acid stimulates the production of growth factors and anti-inflammatory cytokines, for example, interleukin 10 (IL–10). It has the ability to reduce the production of inflammatory cytokines. This is possible due to the ability to inhibit the activation of the nuclear transcription factor kappa B, increased immunogenicity of tumor cells, regulating the activity of proteins involved in apoptosis (Bcl–2, Bak, caspase 3 and 7), increasing the antioxidant activity of glutathione S–transferase (GST), stimulating the production of antibacterial peptides, and inhibiting histone deacetylation and cyclooxygenase (COX)–2. This may affect the silencing of genes involved in the control of cell proliferation, division, and apoptosis [25,48]. 

*Lactobacillus acidophilus*, *Lactobacillus casei*, *Lactobacillus plantarum*, *Lactobacillus delbrueckii*, and *Propionicaterium freuenreichii* have the ability to produce conjugated linoleic acid (CLA) from linoleic acid. It is produced in the distal part of the gut through bacteria. It can be absorbed through or in combination with colonocytes in the lumen of the intestine, thus exerting locally beneficial effects [51].

VSL#3 is a mixture of 8 bacteria belonging to lactic acid bacteria. They affect the strengthening of the epithelial barrier and increase the protein expression of tight junctions. Bassaganaya–Rier et al. investigated whether VSL#3 inhibits intestinal inflammation by altering the diversity of colonic microbes and increasing the production of microbial CLA, which in turn activates the PPAR–γ receptor. It has been shown that changes in the biodiversity of microorganisms and the production of local CLA are associated with the dependent PPAR-γ mechanisms underlying the anti-cancer and anti-inflammatory action of probiotic bacteria [52]. 

In addition, CLA is able to suppress the production of eicosanoids in colonocytes in two ways. Firstly, it replaces arachidonic acid in the cell membranes with linolenic acid. Secondly, it is the result of CLA interference in the activity of lipoxygenase and cyclooxygenase enzymes responsible for the synthesis of eicosanoids. The anticarcinogenic activity of CLA is also dependent on the dose. Consuming probiotic bacteria can increase the production of this fatty acid, which is necessary to promote anticancer activity [25,51].

It is worth noting that bacteria *Pedicoccus petosaceus* 16:1, *Lactobacillus plantarum* 2592 and *Lactobacillus paracasei* F19 produce antioxidants corresponding to 100 mg of vitamin C. Antioxidant capacity may inhibit peroxidation and free radicals to prevent tumor formation [53]. 

Selected probiotic bacteria and their metabolites have been used to promote cell differentiation and reduce DNA damage [28].

The antiproliferative and pro-apoptotic activity of linoleic acid results from its ability to intensify the expression of the gamma receptor (activated by peroxisome proliferates) [25]. The PPAR–γ receptor (Peroxisome proliferator-activated receptor gamma) is involved in the modulation of lipid metabolism. It may also affect cell proliferation, survival, and differentiation. Conjugated linoleic acid affects the expression of genes which are involved in the process of apoptosis and the cellular response to cell growth factors [25,52,54,55].

### 3.4. Improvement of the Physicochemical Condition of Large Intestine

Apoptosis is the genetically programmed cell death of the organism. Reduced ability to induce programmed cell death is an important pathogenic event in many types of cancers. It is accompanied by a change in the control of cell proliferation processes. The regulation of survival as well as cell death with probiotics, for example, can have huge therapeutic and also chemopreventive potential [29].

CRC patients have a disorder of the physicochemical properties of the digestive tract in the intestine, for example, active acidity or viscosity. Probiotics can modify this environment by increasing resistance to cancerogenesis [28]. 

Probiotic bacteria of the genus *Bifidobacterium* and *Lactobacillus* producing lactic, propionic, and acetic acid can lower the intestinal pH and thus inhibit the development of pathogenic bacteria, thus maintaining the balance in the intestines [56]. 

In Lan et al.’s publication from 2007, an acidic environment changed the apoptosis cells in their necrosis. This environment was created by exposing propionibacteria to short-chain fatty acids [57]. Probiotics may be strongly related with the reduction of hypertensivity/bowel irritation of the intestine and pathological changes causing inflammation and abnormal cell growth [28]. 

In addition, the toxicity of water content in the feces and the degree of intestinal water absorption are the first signs of hypersensitivity of the mucous membrane of the colon. Changes in active acidity (a lower pH in the faeces) may block the enzymatic activity of commensal bacteria and their binding to the surrounding epithelial cells [28]. 

### 3.5. Binding and Deactivation of Cancerogenic Compounds. Decreasing the Production of Harmful Enzymes

The ability to bind and deactivate/degrade cancerogenic compounds seems to be strongly connected with the type of bacterial strain, the viability of microorganism, the kind of cancerogenic compound, the probiotic dose and environmental conditions, for example, active acidity, and the presence of gastrointestinal enzymes and bile acids [25]. 

The inadequate composition of the intestinal ecosystem can favour the secretion of bacterial enzymes, such as azoreductase, beta–glucosidase, beta–glucuronidase, and nitroreductase, which affect the production of cancerogenic compounds. They have an impact on creating aromatic amines, aglycone, secondary bile acids, hydrogen sulphide, acetaldehyde, and reactive oxygen species (ROS), for example. Beta–glucosidase can hydrolyze glucuronide, the detoxifying compound, and produce other carcinogens. Probiotics produce substances of an anticancerogenic nature (A3 chromocin, saromycin, neocarcinomycin), as well as dispose procancerogenic compounds (aflatoxins, azo dyes, nitrosamines) [6,28,29,58]. 

The process of conjugation with glucuronic acid is one of the processes that occurs in the liver. As a result of that process, toxic and carcinogenic compounds of exogenous and endogenous origin are inactivated. The emerging conjugates are effectively removed from the body, for example, with bile. A large proportion of the bile salts is reabsorbed in the ileum and then transported through the portal vein back to the liver. What has not been returned to the liver passes into the large intestine, where another transformation occurs (involving intestinal bacteria). Beta–glucuronidase of bacterial origin hydrolyzes everything which gets into the intestine and once again releases carcinogenic aglycons [6,16,29]. 

Bacteria of the genus *Bacteroides, Clostridium, Enterococcus, Salmonella,* and *Staphylococcus* produce enzymes such as nitroreductase and azoreductase, which metabolize dyes, drugs, and aromatic nitro compounds, as a result of which toxic aromatic amines are formed. Moreover, bacteria of the genus *Enterobacter, Enterococcus, Streptococcus, Citrobacter,* and *Escherichia* increase the activity of alcohol dehydrogenases and the production of acetaldehyde (a carcinogen) [28]. 

The relative risk of developing colorectal cancer occurs in people with an increased level of red meat consumption compared to people who consume meat in smaller quantities. This may be due to the presence of heterocyclic aromatic amines (HCA) formed as a result of cooking foods at high temperatures. The intestinal microbiota can activate HCA to the active mutagenic form and it can affect the mucous membranes of the large intestine, causing cancer mutations. Detoxification of mutagenic compounds from cooked food may be one of the mechanisms by which probiotics may decrease the incidence of CRC. It was found that LAB and commensal bacteria may bind and metabolize some of the carcinogens, such as HCA or N–nitro compounds. These properties are associated with a decrease in the mutagenicity that has been observed after exposure of HCA to various bacterial strains [29,59]. 

Regular uptake of probiotic bacteria can decrease pathogenic bacteria in the gut and, consequently, reduce the production of carcinogenic compounds [25]. 

Lactic acid bacteria are able to reduce the activity of carcinogenic compounds 1,2–dimethylhydrazine (DMH), N–methyl–N’–nitro-N–nitrosoguanidyne (MNNG), by uptaking reactive intermediates, producing compounds that deactivate carcinogens and antioxidant enzymes, for example, superoxide dismutase (SOD), glutathione S–transferase (GST), glutathione reductase (GR), glutathione peroxidase (GPx), and catalase (CAT) [15,28,60]. Walia et al. showed that administration of DMH to rats reduced the activity of GPx, GST, SOD, CAT, and GSH (glutathione). In contrast, administration of a chemotherapeutic with probiotics (*Lactobacillus plantarum* (AdF10)) and *Lactobacillus rhamnosus* GG (LGG)) significantly increased the activity of the enzymes mentioned above. This suggests that probiotics may protect against oxidative stress during treatment of induced colon cancer [60].

### 3.6. Reducing Inflammation in the Intestine 

Probiotic bacteria contribute to the proper functioning of the immune system. They can suppress and also improve the intestinal and systemic immune response. They contribute to the differentiation of immune system cells, for example, dendritic cells, T and B lymphocytes. By stimulating the production of anti-inflammatory substances, antioxidants, and anti-cancer components, probiotics affect the cellular and immune response. Modification of the expression of cytokine proteins creates a wide range of probiotics in the treatment of the prevention of diseases associated with abnormal immune systems [28,61,62,63]. 

Potentially, this is due to the release of bacterial products such as proteins (flagellin), LPS (lipopolysaccharides) and the fact that probiotic bacteria [28,61,62,64,65]:

- interact with TLR (Toll-like) receptors. TLRs play an important role in the initiation of the immune response, as well as the recognition of the threat,
- affect the production of IL–8 (interleukin 8) needed for neutrophilia,
- induce the production of anti-inflammatory cytokines, inhibit NF–kB in macrophages, and initiate the production of TNF (tumor necrosis factor, which is a pleiotropic cytokine involved in the pathogenesis of many physiological processes that control inflammation or antineoplastic response etc.),
- prevent the activation of the NF–kB transcription factor, which plays a key role in activating the immune system to various stimuli
- modify the MAPK kinase (mitogen-activated protein kinases) signaling pathway and the PPAR–γ receptor.

Many strains of *Lactobacillus* bacteria induce IFN–γ (interferon–γ) and IL–12 secretion through Th1 cells. They are cytokines related to cellular immunity. However, they can also affect the secretion of IL–4 (interleukin–4) and IL–5 (interleukin–5) cytokines through Th2 lymphocytes, which in turn stimulates a humoral response [66].

Short-chain fatty acids have immunomodulatory functions that affect the inflammatory response in selected cases by interacting with receptors that are coupled to G protein in the intestine. G protein is a heterotrimeric guanidine nucleotide binding protein. It is likely that there is no single cell in the body in which G protein would not participate in the transmission of signals. Neurotransmitters or chemokines transmit cell signals with the participation of G protein [67,68,69]. The immune system of the host plays an important role in controlling tumor promotion, as well as its progression. The interaction of many elements of the immune system, antigen presenting cells (APC), T cells, B cells, and NK cells is extremely important. These cells are necessary for the production of an anti-cancer immune response [29]. 

It is noted that *Lactobacillus fermentum* NCIMB 5221 shows increased antiproliferative activity against colorectal cancer as well as producing more free fatty acids (FFA) compared to *Lactobacillus acidophillus* ATCC 314 and *Lactobacillus rhamnosus* ATCC 53103. *Lactobacillus fermentum* NCIMB 5221 also produces more butyric and acetic acid. It is the only bacterium that produces propionic acid [10]. 

In the presence of butyrate, macrophages reduce the secretion of pro-inflammatory mediators induced by LPS (inter alia, IL–6 (interleukin–6) or IL–12) but have no effect on TNF–α, for example [70]. 

Anti-inflammatory effects are also exhibited by propionic and acetic acid. These compounds have the ability to inhibit the activation of the transcription kappa B factor, as well as regulate the expression of proinflammatory cytokines. Propionic acid can stimulate tumor cell apoptosis and regulate the expression of proinflammatory cytokines. After butyric acid, propionic acid is the second short-chain acid used by colonocytes as an energy source [25]. 

*Lactobacillus acidophilus, Lactobacillus casei*, and *Bifidobacterium* spp. affect the amount of gamma-interferon in the blood, and stimulation the proliferation and also activity of T and B lymphocytes. B lymphocytes produce immunoglobulin A, which, by preventing intestinal colonization through undesirable microbiota, protects the intestinal epithelium [9]. 

## 4. A Potential Dose of Probiotic Therapy Can Bring Health Benefits 

The results of the study indicate that enriching the diet with 10^9^–10^12^ bacterial cells with a probiotic effect, even after a few weeks of consumption, can lead to an increase in the activity of macrophages, as well as of lymphocytes, interferon gamma, and immunoglobulin in the blood serum [8,16,71]. 

It is believed that the consumption of bacteria from the *Lactobacillus* and *Bifidobacterium* genus at a dose of 10^10^–10^11^ CFU/per day for a minimum of 4–6 weeks can reduce the risk of colorectal cancer. More research is needed in this area to study the relationship between probiotics, diet, and the risk of cancer [72]. 

Too little data is currently available on the optimal number of viable probiotic bacteria that should be recommended for both treatment and prevention of cancer. This number is difficult to estimate, because it depends on the specific bacterial strain and the benefits that the host has to bring. The total amount of probiotics administered cannot be low if it is to have a successful impact on the host microbiota. As mentioned previously, special attention should also be paid to bacterial competition on the intestinal epithelium, and their number must be high enough to obtain a beneficial effect [72,73].

Due to the lack of data on specific doses to be used in treatment and prevention, attention is drawn to the information contained in the document AFSSA (Aureli’s publication) [73]:

- The number of probiotic bacteria consumed is an important factor to obtain a high concentration of bacteria in various sections of the digestive tract,
- The concentration of probiotics should be greater than or equal to 10^6^ CFU/ml in the small intestine (ileum) and 10^8^ CFU/g in the large intestine (the strength of scientific studies confirming this thesis is weak),
- Concentration in the colon has been proposed because, in justified cases, it can be expected that the bacterial flora is more likely to be more active than the flora present at lower levels.

## 5. The Importance of Probiotics in the Prevention and Treatment of Cancer Tumors

A study conducted by Marteau et al. showed that decreased levels of nitro-reductase after a three-week period of consumption of lactic fermented products containing *Lactobacillus acidophillus, Bifidobacterium bifidum,* and mesophilic *Streptococcus lactis* and *Streptococcus cremoris* cultures did not change the activity of beta–gluonidase and azoreductase. This proves that the ability to modulate the activity of bacterial enzymes is dependent on the probiotic strain [29,74]. 

The study carried out on cell lines showed that LAB has the ability to increase the induction of 5–fluorouracil apoptosis (5–FU), a chemotherapeutic agent, as well as having a synergistic effect with the agent [75]. A drug that is used during chemotherapy, 5–fluorouracil (5–FU), often affects the occurrence of diarrhea. Researchers compared two treatments based on 5–FU, and the effect of Lactobacillus supplementation and fiber on treatment tolerance. Patients diagnosed with colon cancer (*n* = 150) were randomly assigned to two groups for 24 weeks (this was complementary therapy). The study group received *Lactobacillus rhamnosus* GG supplementation (1–2 × 10^10^ a day) in addition to 11 g of guar gum during chemotherapy (the second group did not receive a probiotic and guar gum during this stage of treatment). Patients who received a probiotic did not have acute diarrhea (fewer than 4) and also reported less discomfort in the abdominal cavity. It should be noted that patients receiving this bacterial strain required shorter hospital care and received lower doses of chemotherapy compared patients without any intake of these bacteria [76]. 

It was also observed that the administration of *Bifidobacterium breve* Yakult to patients during chemotherapy protects them against infections and modification of the intestinal ecosystem [77]. 

In another study with 206 radiotherapy patients, the administration of *Lactobacillus rhamnosus* alleviated gastrointestinal toxicity associated with radiation [78]. 

The administration of *Lactobacillus acidophilus* and *Bifidobacterium bifidum* also resulted in a significant improvement in stool consistency, a reduction in radiotherapy-induced diarrhea, and reduced the need for anti-polar agents [79]. 

Research related to the effect of probiotic bacteria on cancer cells, and also to animals with induced cancer or having administered carcinogens, is presented in Table 2. These results show that probiotics have anti-cancer properties.

## 6. Probiotics and Operations

Clinical studies have shown that some probiotic strains can be helpful in controlling postoperative inflammatory conditions [88]. 

*Lactobacillus johnsoni* La1, administered orally before and after the treatment, adheres to the intestinal mucosa, reducing the number of potentially pathogenic bacteria in the faeces (enterobacteria and enetorococci) and modulating local immunity [89].

Infection during abdominal surgery, which is considered as a factor affecting the morbidity of patients, can be reduced by administering probiotic bacteria to patients prior to their operation. In the study by Liu et al., 150 people were randomly assigned to two groups, of which one received a supplement and the other did not. The number of postoperative infections in the group using the probiotic was lower than in the control group. Postoperative sepsis occurred in a smaller number of people in the group using the probiotic supplement (41 cases), compared with the control group (55 cases) [90].

Similar conclusions were drawn by the researchers in an earlier study [91]. 

Fermented dairy products, which have been suggested as products affecting the human body, protect against the occurrence of colorectal cancer. The prospective cohort study by Pala et al. was conducted for approximately 12 years. At that time, out of the 45,241 patients examined, 289 patients were diagnosed with colon cancer. This study showed that consumption of yogurt is associated with a significant reduction in the risk of colorectal cancer. Therefore, fermented products should be part of the diet [92]. 

Studies on humans related to the use of probiotics for prophylaxis, as well as in the treatment of colorectal cancer, have been included in Table 3. It has been shown, among other things, that perioperative administration of probiotics effectively reduces post-operative infectious complications.

## 7. Conclusions

Based on the literature review, it can be concluded that probiotics can exert an influence both locally and on the body as a whole. Through the mechanisms presented in this paper, probiotics can support both prevention and treatment of colon cancer. However, their effect depends on the bacterial strain and thus on the properties it exhibits. Therefore, it is important to continue research on probiotics (especially their properties), and the mechanisms through which they act (especially during treatment). There is great potential for *Lactobacillus fermentum* NCIMB 5221, inter alia.

## Figures and Tables

**Table 1:** The amount and types of microorganisms in the gastrointestinal tract (GIT) of human [17,20,21,22,23].

Gastrointestinal Tract	Total Colonic Number (log CFU/mL)	Main Types of Microorganisms
Oral cavity	10^8^	*Streptococcus, Eubacteria, Capnocytophaga, Veillonella, Fusobacterium, Porphyromonas, Prevotella, Neisseria, Treponema, Lactobacterium, Eikenella, Leptotrichia, Peptostreptococcus, Propionibacterium, Rothia, Scardovia, Parascardovia, Alloscardovia, Candida, Saccharomyces, Penicillium, Scopularis, Aspergillus, Fusarium, Cryptococcus, Alternaria, Geotrichum*
Oesophagus	10^4–6^	*Streptococcus, Prevotella, Veillonella*
Stomach	10^2–4^	*Helicobacter (species pylori), Lactobacillus, Staphylococcus, Streptococcus, Clostridium, Capnocytophaga, Deinococcus, Veillonella, Escherichia, Bifidobacterium, Prevotella, Caulobacter, Actinobacillus, Corynebacterium, Rothia, Gemella, Leptotrichia, Porphyromonas*
Duodenum	10^3^	*Enterococcus, Lactobacillus, Bacteroides, Bifidobacterium, Clostridium, Enterobacteriaceae, yeast*
Jejunum	10^4^	
Ileum	10^7^	
Large intestine	10^10–11^	*Enterococcus, Lactobacillus, Bacteroides, Fusobacterium, Bifidobacterium, Clostridium, Enterobacteriaceae, Peptococcus, Peptostreptococcus, Staphylococcus, Ruminococcus, Eubacterium, Streptococcus, Actinomyces, Finegoldia (species magna), Micromonas (species micros), Peptococcus (species niger), Veillonella, Escherichia (species coli), Klebsiella, Proteus, Pseudomonas, Enterococcus (species faecalis), Bacillus*
Rectum	10^11–12^	

**Table 2:** The impact of probiotic bacteria on cancer cell lines and on animals with induced colorectal cancer.

**RESEARCH ON CELL LINES/*IN VITRO***			
**Probiotic Bacteria**	**Cell Lines**	**Effects/Mechanisms**	**Source**
*Lactobacillus rhamnosus GG*	Caco–2	Decreased level of IL–8.	LOPEZ et al. 2008 [80]
*40 different probiotic bacteria isolates*	Caco–2, HRT–18 Vero cells Using Trypan Blue assays (TBE) and 3–(4, 5–dimethylthiazolyl–2)–2, 5–diphenyltetrazolium bromide (MTT)	Two isolates of *Lactobacillus acidophilus* LA102 and *Lactobacillus casei* LC232 showed clear cytotoxic activity. They showed no cytotoxic activity on normal Vero cells.	AWAISHEH et al. 2016 [81]
*Lactobacillus casei* ATCC393	CT26 (murine colon carcinoma cell lines); HT29 (human colon carcinoma cell lines) Administration of live *L. casei* and bacterial components to cell lines.	Anti-proliferative activity. Live *L. casei* induced apoptotic death of CT26 and HT29 cells.	TIPTIRI-KOURPETI et al. 2016 [82]
**RESEARCH ON ANIMAL MODELS/*IN VIVO***			
**Probiotic Bacteria**	**Animal Models**	**Effects/Mechanisms**	**Source**
*Bacillus polyfermenticus*	Five-week-old male F344 rats. Three research groups two of which were administered DMH (one was the control group, the other the study group).	Reduction in the formation of ACF (aberrant crypt foci) of about 50%, in the group with supplementation of *B. polyfermenticus*. Increased of antioxidant potential.	PARK et al. 2007 [83]
*Lactobacillus plantarum*	Six-month-old male and female Wistar albino rats with induced colon cancer with DMH.	Reduced concentration of bile acid and bacterial enzymes. Increased level of TNF-alpha in the serum and the number of bacteria of the *Lactobacillus* genus.	BERTKOVA et al. 2010 [84]
*Lactobacillus acidophilus, Lactobacillus casei* and *lactis* biotype *diacetylactis* DRC-1	Rats DMH-induced CRC model. 100 rats were divided into four groups (DMH control group, probiotic curd group, normal curd group, and normal diet group).	Decreasing the incidence, number and size of tumors. Significant reduction in DNA damage.	KUMAR et al. 2010 [85]
*Lactobacillus rhamnosus* 231 (Lr 231)	Male Wistar rats exposed to MNNG (N–Methyl–N’–Nitro–Nitrosoguanidine).	Decreased fecal activity of azoreductase, nitroreductase, GST. Increased GSH.	GOSAI et al. 2011 [40]
*Lactobacillus acidophilus* KFRI342 (isolated from kimchi)	Forty-five male F344 rats with DMH chemically induced premalignant lesions in the colon.	Reduction in ACF, beta-glucuronidase, beta-glucosidase activity, decreased intestinal population of aerobic bacteria and *Escherichia coli* (in stool samples).	CHANG et al. 2012 [86]
*Lactobacillus casei* BL23	C57BL/6 mice 1,2–dimethylhydrazine (DMH) was injected subcutaneously every week for 10 weeks. L. casei BL23 was also administered orally in drinking water for up to 10 weeks.	Modulation of host immune response. *L. casei* BL23 protect mice against DMH-induced colorectal cancer.	LENOIR et al. 2016 [87]

**Table 3:** The results of the impact of probiotic bacteria in the prevention and in the treatment of colon cancer.

Research on Human			
Prevention			
Probiotic Bacteria	Subjects	Effects/Mechanisms	Source
*Lactobacillus rhamnosus* LC705 and *Propionibacterium freudenreichii* ssp. *shermanii* JS	38 men (between 24 and 55 years old).	Decreased beta-glucosidase activity (by 10%) and urease (by 13%). Increasing the fecal amount of bacteria of the genus *Lactobacillus* and propionibacteria.	HATAKKA et al. 2008 [93]
*Lactobacillus gasseri* OLL2716 (LG21)	10 people with colorectal cancer and 20 healthy patients	Increasing the number of bacteria from the genus *Lactobacillus,* synthesis of isobutyric acid, NK cell activity. Reducing the amount of *Clostridium perfringens*.	OHARA et al. 2010 [94]
*Streptococcus thermophilus* and *Lactobacillus delbruckii* subsp. *bulgaricus*	45 241 healthy people (14 178 men, 31 063 women)	Reduction in the risk of colorectal cancer correlated with increased consumption of yogurt (especially in men).	PALA et al. 2011 [92]
TREATMENT			
*Bifidobacterium longum*	60 patients with colorectal cancer undergoing colon resection	Increasing the amount of bacteria of the genus *Bifidobacterium*, and reducing the amount of bacteria of the genus *Escherichia* ratio of these bacteria was different to the pre-operative.	ZHANG et al. 2012 [95]
*Bifidobacterium breve* strain Yakult	42 patients during chemotherapy (19 people were in the study group, 23 in the control group)	Reduction in the incidence of fever and the use of intravenous antibiotics was lower in the study group than in the control group.	WADA et al. 2010 [77]
*Lactobacillus acidophilus*, *L. plantarum*, *Bifidobacterium lactis* and *Saccharomyces boulardii*	164 patients with colorectal cancer undergoing colorectal surgery	Significantly decreased the risk of postoperative complications. In the probiotic group, a positive correlation was observed between the expression of the SOCS3 gene and the expression of the TNF gene and circulating IL–6.	KOTZAMPASSI et al. 2015 [96]
